# Performance demonstration of a hybrid Compton camera with an active pinhole for wide-band X-ray and gamma-ray imaging

**DOI:** 10.1038/s41598-020-71019-5

**Published:** 2020-08-20

**Authors:** Akihisa Omata, Jun Kataoka, Kazuya Fujieda, Shogo Sato, Eri Kuriyama, Hiroki Kato, Atsushi Toyoshima, Takahiro Teramoto, Kazuhiro Ooe, Yuwei Liu, Keiko Matsunaga, Takashi Kamiya, Tadashi Watabe, Eku Shimosegawa, Jun Hatazawa

**Affiliations:** 1 Graduate School of Advanced Science and Engineering, Waseda University, Tokyo, Japan; 2 Graduate School of Medicine, Osaka University, Osaka, Japan; 3 Institute for Radiation Science, Osaka University, Osaka, Japan

**Keywords:** Imaging techniques, Experimental nuclear physics, Molecular medicine, Cancer imaging

## Abstract

X-ray and gamma-ray imaging are technologies with several applications in nuclear medicine, homeland security, and high-energy astrophysics. However, it is generally difficult to realize simultaneous wide-band imaging ranging from a few tens of keV to MeV because different interactions between photons and the detector material occur, depending on the photon energies. For instance, photoabsorption occurs below 100 keV, whereas Compton scattering dominates above a few hundreds of keV. Moreover, radioactive sources generally emit *both* X-ray and gamma-ray photons. In this study, we develop a “hybrid” Compton camera that can simultaneously achieve X-ray and gamma-ray imaging by combining features of “Compton” and “pinhole” cameras in a single detector system. Similar to conventional Compton cameras, the detector consists of two layers of scintillator arrays with the forward layer acting as a scatterer for high-energy photons (> 200 keV) and an active pinhole for low-energy photons (< 200 keV). The experimental results on the performance of the hybrid camera were consistent with those from the Geant4 simulation. We simultaneously imaged $^{241}$Am (60 keV) and $^{137}$Cs (662 keV) in the same field of view, achieving an angular resolution of 10$^\circ$ (FWHM) for both sources. In addition, imaging of $^{211}$At was conducted for the application in future nuclear medicine, particularly radionuclide therapy. The initial demonstrative images of the $^{211}$At phantom were reconstructed using the pinhole mode (using 79 keV) and Compton mode (using 570 keV), exhibiting significant similarities in source-position localization. We also verified that a mouse injected with 1 MBq of $^{211}$At can be imaged via pinhole-mode measurement in an hour.

## Introduction

In the field of nuclear medicine, it is essential to visualize the distribution of radioisotopes in a patient’s body. Particularly, a radiological diagnosis that enables non-invasive visualization of the pathology from outside the body is important. The general approach is to visualize nuclear gamma rays emitted from radioactive tracers. Two common techniques—single photon emission computed tomography (SPECT) and positron emission tomography (PET)—play important roles in the diagnosis. However, they image a specific energy range of either X-rays or gamma rays; SPECT can image gamma rays with energies less than 300 keV with the use of the collimator, whereas PET can image positron emitters that emit 511-keV gamma rays. These lead to a limited number of radioactive tracers that can be imaged only with current SPECT and PET scanners. In this context, a Compton camera^1,2^ that can conduct imaging in a wide energy band is reckoned to cause a breakthrough in nuclear medicine^3,4^. Attempts are being made to develop Compton cameras aiming the image of prompt gamma rays emitted from the patient’s body during proton therapy^5–8^. In addition, several works have aimed to realize tracer visualization using the Compton camera. For example, Fontana et al. (2017) describe the Compton camera as an electronically collimated SPECT device, which is, however, optimized for higher energies (above a few hundreds of keV)^9^. Yoshida et al. (2020) proposed whole gamma imaging (WGI) wherein a conventional PET scanner is converted by inserting an additional scatterer to create a Compton camera^10^. They succeeded in realizing the triple gamma (i.e., PET/Compton) imaging of $^{44}$Sc, which emits 511 keV and 1,157 keV gamma rays; however, the application to imaging SPECT tracer remains to be researched in the future. In this context, the simultaneous capture of SPECT and PET images has also been reported using a Compton camera consisting of Si/CdTe semiconductors, which is, however, limited both in terms of detection efficiency and angular resolution^11–13^. We argue that such difficulties can be easily overcome with the hybrid camera proposed in this paper. This increases the variety of radioactive tracers available for nuclear medicine^14–18^. The emergence of new tracers may solve current problems, such as manufacturing costs of tracers, in addition to achieving improvements in the diagnosis quality. A Compton camera also enables the simultaneous imaging of multiple tracers; this significantly increases the information obtained from living organisms in a single diagnosis.

Moreover, nuclear medicines involving radioactive sources are applied not only in the diagnosis but also in the treatment of diseases such as cancer. Particularly, radionuclide therapy (RNT)^19,20^, which uses the targeted radionuclide by administering radioisotopes to patients, is widely used because it damages cancer cells while limiting the exposure of healthy tissues to radiation. For instance, RNT, with $\alpha$ particles, is receiving significant attention because of its high therapeutic potential owing to the higher ionization power for damaging cancer cells^21,22^. Nevertheless, once administered into the body, it is difficult to determine the distribution and pharmacokinetics of a radionuclide. For the safety and effectiveness of RNT, it is conceivable to visualize the characteristic X-rays and nuclear gamma-rays emitted simultaneously with the alpha decay. Some of these characteristic X rays and nuclear gamma rays can be visualized by SPECT. Furthermore, the use of a Compton camera for the in vivo visualization of $^{223}$Ra, an $\alpha$-particle emitter used for RNT, was suggested in our previous study^23^. However, these radionuclides emit several X-rays and gamma rays with different branching ratios. Some emit strong characteristic X-rays that can be imaged with a SPECT, whereas others emit high-energy gamma rays that can be imaged only with a Compton camera (see, Table 1). Imperatively, a simple and cost-effective imaging system that is sensitive to both X-rays and gamma rays is highly desired.

This paper proposes a “hybrid” Compton camera that realizes simultaneous wide-band imaging from a few tens of keV to approximately an MeV, combining some features of “Compton cameras” and “pinhole cameras” in a single detector system. Although the hybrid camera consists of two layers of position-sensitive detectors, similar to a Compton camera^13,23^, the front detector has a hole in the center. Compton and pinhole imaging are enabled using the front detector as a scatterer for high-energy photons (> 200 keV) and an active pinhole for low-energy photons (< 200 keV). We developed a prototype of the hybrid camera. Simulation and experimental results depicted resolutions better than 10$^{\circ }$ (full width at half maximum (FWHM)) in the range of 50–1,000 keV. In addition, the imaging of $^{211}$At is receiving attention as a source applicable to RNT with $\alpha$-particles^24–27^. We initially investigated the capability of our hybrid camera system with a simple phantom of $^{211}$At and thus conducted mouse imaging.

**Figure 1:**
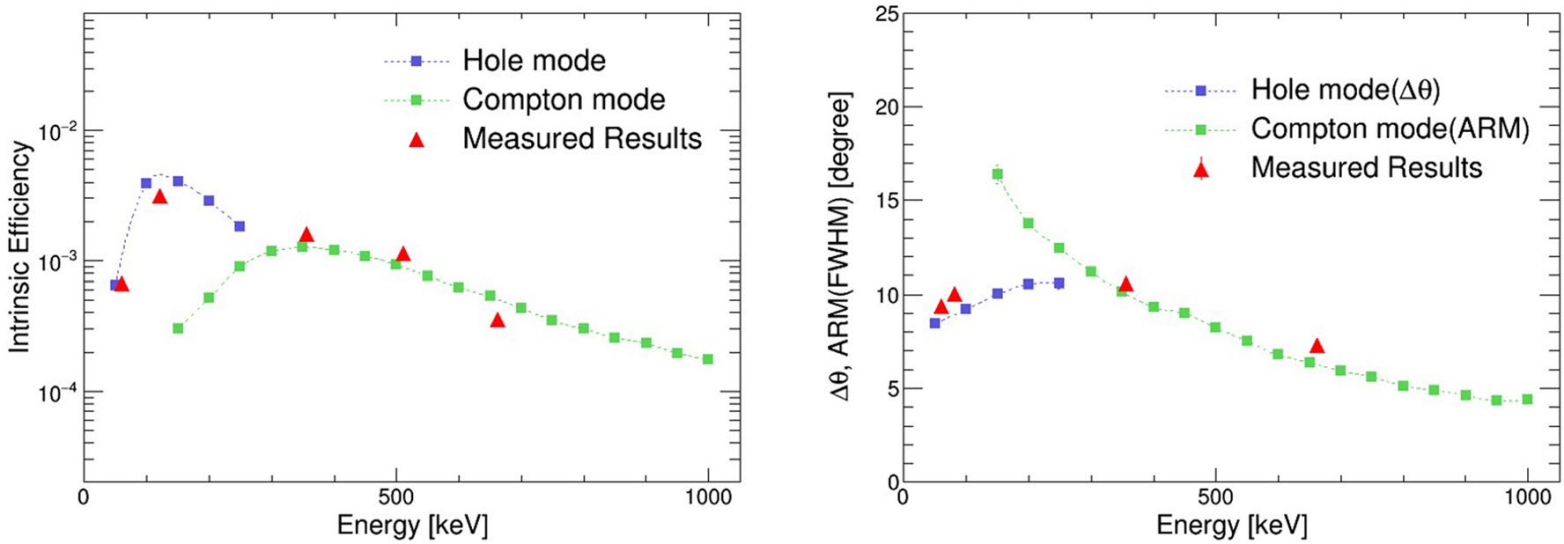
Comparison of the simulated energy response of the intrinsic efficiency (left) and angular resolution (right) with the results of actual measurements.

## Results

### Performance evaluation

The imaging performance of the hybrid camera was initially evaluated by simulations using Geant4^28^. The simulation configuration includes the scintillator arrays, the MPPC arrays, and the metal case, which are detailed in the “Methods” section. A monochromatic point source placed 30 cm from the camera and at the center of the field of view (FOV) was imaged. The detector performance was investigated between 50 and 1,000 keV for both the pinhole and Compton modes. Considering the energy and spatial resolution of the actual detectors, we utilized a reconstruction flow as described in the “Methods” section. The simulation showed that pinhole imaging is difficult over 250 keV, whereas Compton imaging is active at approximately 150 keV or higher. Figure 1 (left) details the intrinsic detection efficiency that indicates the proportion of events detected either as the pinhole or the Compton mode to all radiation emitted towards the detector. The intrinsic detection efficiency ($\epsilon _{{ int}}$) is expressed by the following equation, using the absolute detection efficiency ($\epsilon _{{ abs}}$):

1 $$\begin{aligned} \epsilon _{{ int}}=\frac{4\pi }{\Omega }\epsilon _{{ abs}} \end{aligned}$$

where $\Omega$ denotes the solid angle of the detector viewed from the source. Figure 1 (right) shows the angular resolution of the pinhole and Compton images as a function of energy. For Compton imaging, we use the angular resolution measure (ARM) that is commonly used to measure the angular resolution of Compton cameras. For measuring the angular resolution of pinhole imaging, the $\Delta \theta$ value is obtained by geometrically converting the position resolution of the image to angular resolution. Note that for the source placed at the center of the FOV, the angular resolution ($\Delta \theta$) can be calculated as follows:

2 $$\begin{aligned} \Delta \theta =\arctan (\Delta x/2l) \end{aligned}$$

where $\Delta x$ and *l* denote the position resolution (FWHM) of the pinhole image and the distance between the camera and the source, respectively. The angular resolution was better than $10^\circ$ in the range of 50–1,000 keV, exhibiting the higher efficiency of the proposed hybrid camera than those of the conventional cameras.

Subsequently, the fundamental imaging performance of the hybrid camera was evaluated by performing experiments under the same geometry as in the simulation. The measurements were recorded at energies of 60 keV and 81 keV for pinhole imaging and 356 keV and 662 keV for Compton imaging. The obtained angular resolutions were consistent with the values predicted by the simulation, as represented by the red plots in Fig. 1 (right).

**Figure 2:**
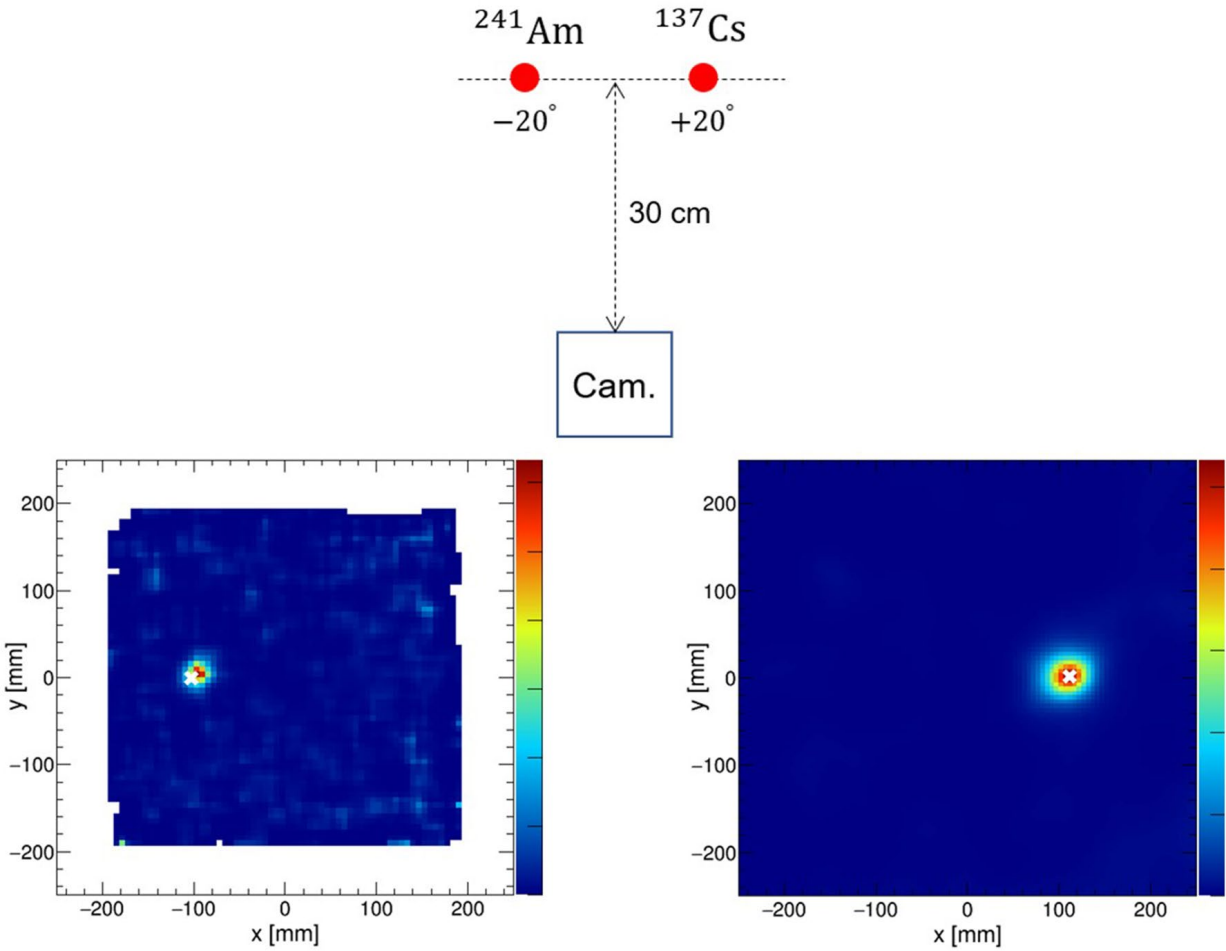
(Upper) The experimental configuration of the simultaneous measurement of $^{241}$Am and $^{137}$Cs. (Lower) The MLEM reconstructed images of the $^{241}$Am (60 keV) source analyzed by the pinhole mode (left) and the $^{137}$Cs (662 keV) source analyzed by the Compton mode (right).

**Figure 3:**
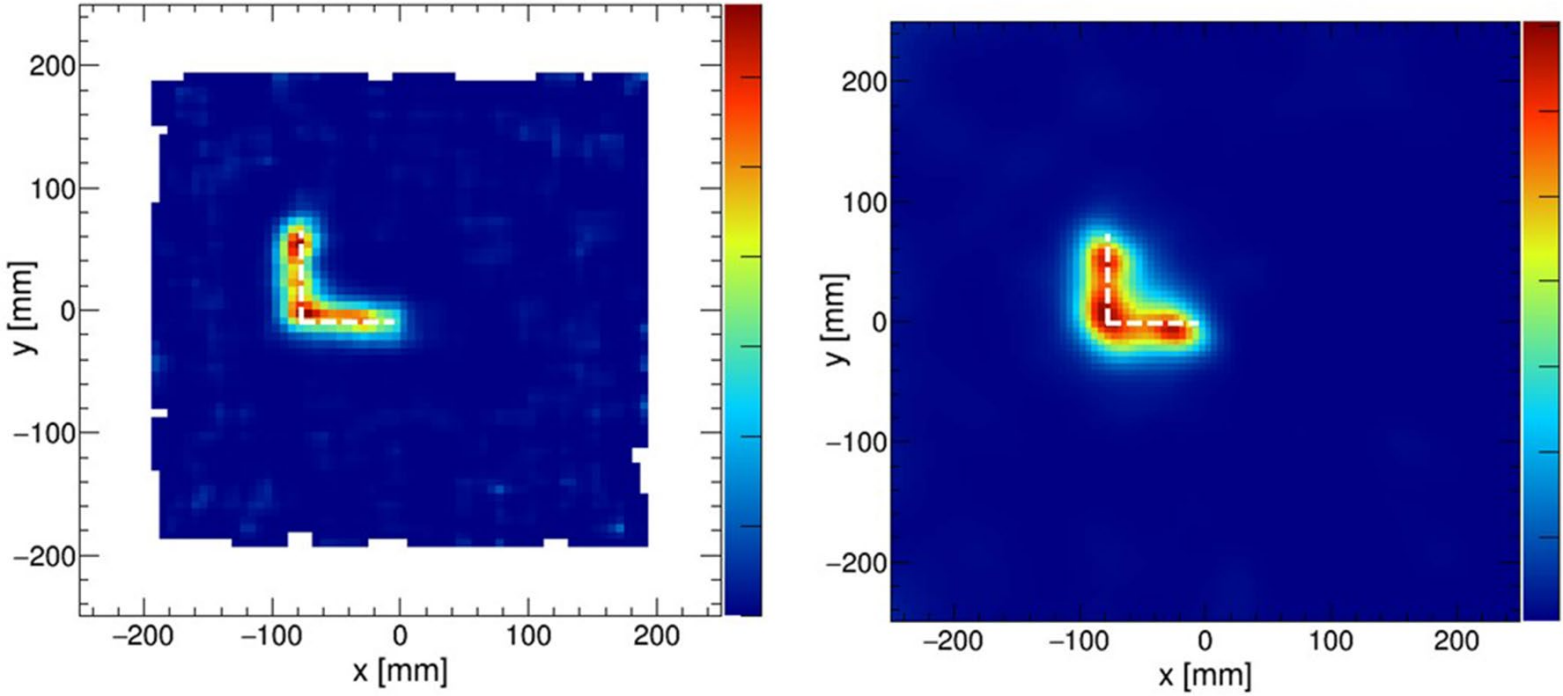
The MLEM reconstructed images of “L”-shaped sources. Pinhole reconstruction of the $^{241}$Am source (left) and Compton reconstruction of the $^{137}$Cs source (right) that were measured separately.

### Experimental demonstration of wide-band imaging

To examine the performance of the hybrid camera, we conducted simultaneous imaging of $^{241}$Am and $^{137}$Cs sources. As shown in Fig. 2 (upper), these sources were placed 30 cm away from the camera and at $+\,20^\circ$ and $-\,20^\circ$, respectively, from the center of the FOV. The $^{241}$Am source was reconstructed in the pinhole mode (60 keV), whereas the $^{137}$Cs source was reconstructed in the Compton mode (662 keV). From Fig. 2 (lower), each convergence indicated the correct positions, depicting the potential of broadband imaging using the hybrid camera.

As a next step, extended sources were measured to examine the validity of the camera system, including the image reconstruction technique. The extended 8 mm $\times$ 8 mm “L”-shaped sources were reproduced by moving on a stage that automatically moved at a constant speed (0.96 mm/min). The image of an “L”-shaped $^{241}$Am source was reconstructed by the pinhole mode using events with energies around 60 keV. Subsequently, the image of an “L”-shaped $^{137}$Cs source (662 keV) was also reconstructed by the Compton mode. MLEM reconstruction images of each measurement are presented in Fig. 3.

**Figure 4:**
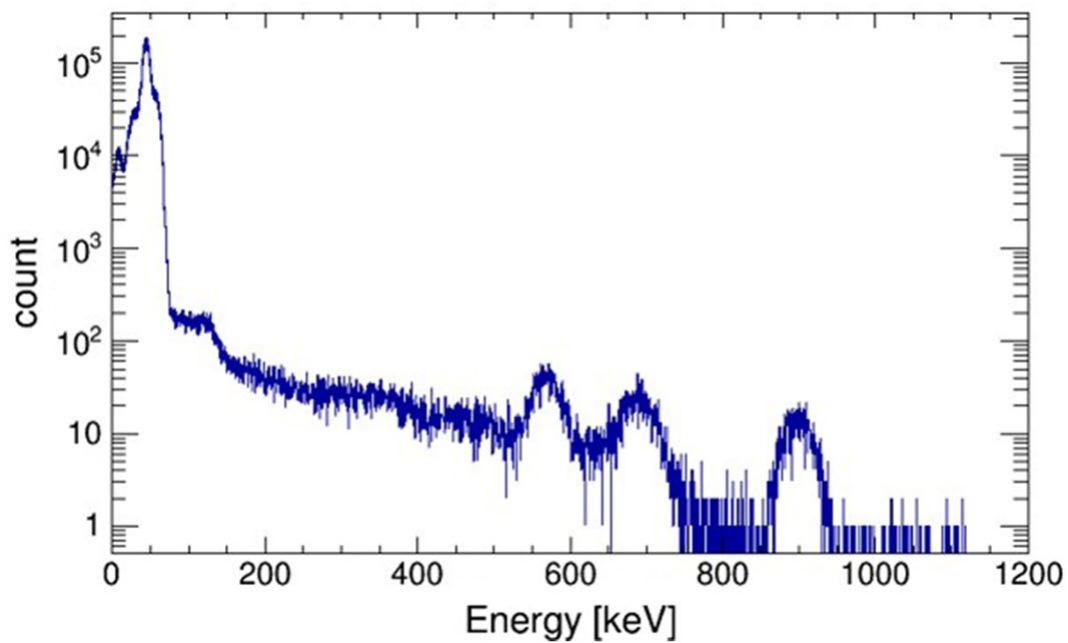
Energy spectrum of $^{211}$At obtained by a LaBr$_3$ scintillator coupled to a PMT.

**Figure 5:**
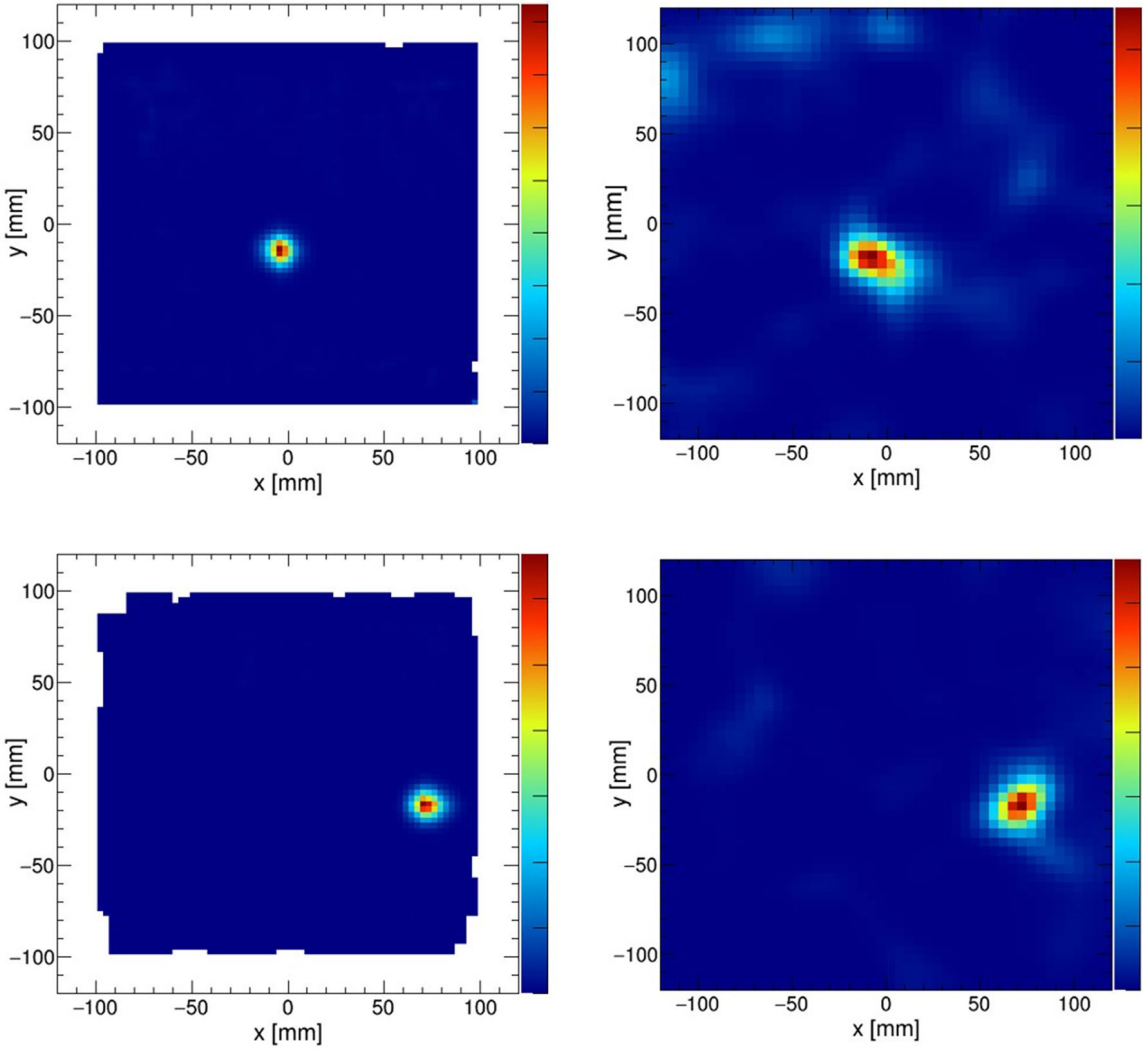
The pinhole MLEM reconstruction image (left) and the Compton MLEM reconstruction image (right) of a bottle with $^{211}$At at the center of the FOV (upper) and $30^\circ$ to the right (lower).

### $^{211}$At imaging of a small bottle

Furthermore, we investigated the performance of the hybrid camera with the imaging of a $^{211}$At source, which is an $\alpha$-particles source for RNT. Initially, the energy spectrum of $^{211}$At was obtained by a LaBr$_3$ scintillator coupled to a photomultiplier tube (PMT). Figure 4 shows peaks of characteristic X-rays (mainly 79 keV) and gamma rays with energies of 570, 687 and 898 keV. Furthermore, the intensity of X-rays is approximately three orders of magnitude higher than that of the gamma rays.

Next, placing 15 cm away from the hybrid camera at $0^\circ$ (the center of the FOV) and $30^\circ$ to the right, a small bottle was imaged with 400 μL of $^{211}$At (4.61 MBq). The measurement times were 20 and 45 min at $0^\circ$ and $30^\circ$, respectively. The images were reconstructed using 79 keV X-rays in the pinhole mode and 570 keV gamma rays in the Compton mode. The measurements resulted in 32,822 pinhole events and 107 Compton events at $0^\circ$ and 32,710 pinhole events and 119 Compton events at $30^\circ$. The pinhole images of the 79 keV X-rays and the Compton images of the 570 keV gamma rays are presented in Fig. 5.

**Figure 6:**
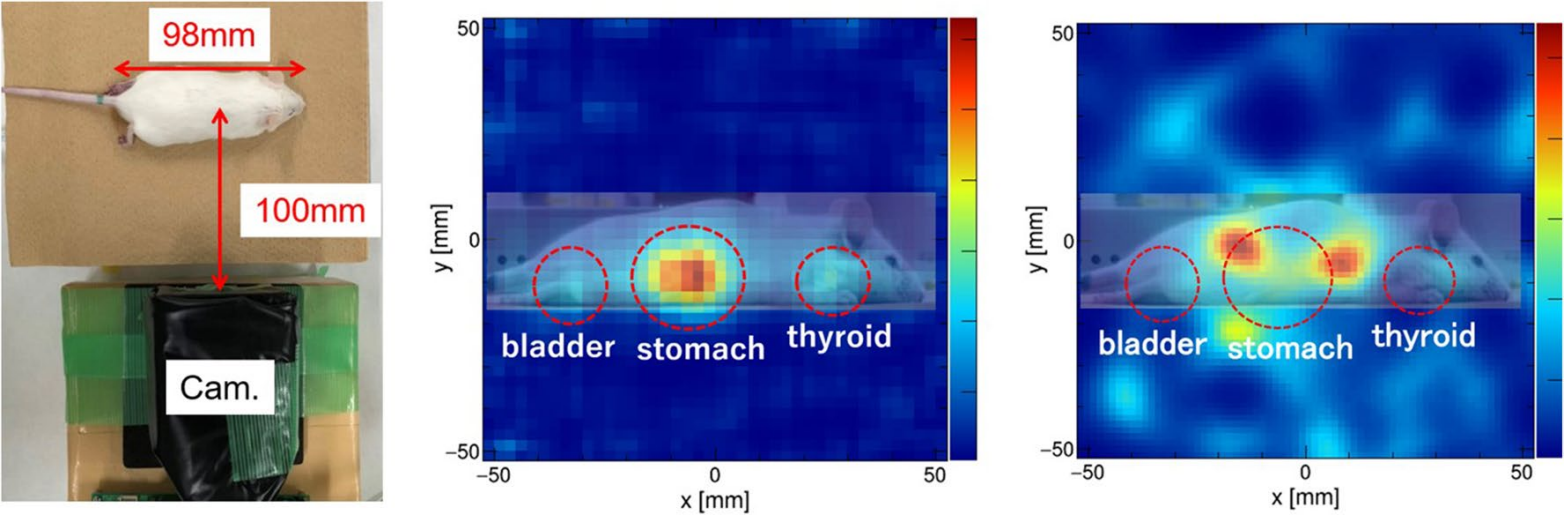
(Left) Experimental configuration of the measurement of the mouse administered with $^{211}$At. (Center) The pinhole MLEM reconstructed image obtained by 1 h of measurement. (Right) The Compton MLEM reconstructed image obtained by 11 h of measurement.

### $^{211}$At imaging of a mouse

To investigate the capability of the hybrid camera for animal imaging, imaging of a mouse [8-week-old male ICR mouse (SLC Japan, Hamamatsu, Japan)] with a $^{211}$At tracer was conducted. The mouse (length 98 mm, weight 39.3 g) was injected with $^{211}$At (971 kBq) 10 h before the measurement. The mouse was euthanized by an overdose of isoflurane 3 h after the injection. The hybrid camera was positioned on the right side of the mouse, 100 mm from the body axis, as shown in Fig. 6 (left). The measurement time was 11 h, resulting in 79,969 pinhole events and 243 Compton events. From Fig. 6 (center), the pinhole image depicted that the distribution of the $^{211}$At converged on the thyroid, stomach, and bladder from 11,755 events obtained after 1 h of measurement. Moreover, although the Compton image confirms the concentration near the center, accumulation is splitting, possibly owing to the lack of event statistics, as shown in Fig. 6 (right). All the animal experiments were approved by the animal ethics committees of Osaka University and were performed according to the institutional guidelines.

**Table 1:** Examples of $\alpha$-particle emitters for RNT and their main X-rays and gamma rays (and their absolute intensities, in percentage) accompanied by the adaptability of the pinhole mode and/or the Compton mode.

Radionuclide	X ray (keV)	Gamma ray (keV) (absolute intensity)	Pinhole	Compton
$^{211}$At	79 (31.1%)	570 (0.3%)	$\checkmark$	
$^{213}$Bi	79 (3.2%)	440 (26.1%), 465 (2.1%), 1,567 (2.2%)		$\checkmark$
$^{225}$Ac		218 (11.6%), 440 (26.1%)		$\checkmark$
$^{223}$Ra	83 (41.7%)	351 (12.9%)	$\checkmark$	$\checkmark$
$^{212}$Bi		239 (43.3%), 511 (8.2%), 583 (30.4%), 2,615 (35.7%)		$\checkmark$

## Discussion

In the imaging experiment with the $^{211}$At inside a small bottle, the source position was obtained by pinhole reconstruction using the events accumulated in the first 5 s. The time taken to localize the convergence through the pinhole mode was 1/100 of that thorough the Compton mode. This is due to the intense X-ray emission from the $^{211}$At, as shown in Fig. 4. 79 keV X-rays are statistically advantageous over 570 keV gamma rays for imaging $^{211}$At. In this regard, it is comprehensible that Compton events are not enough to reconstruct the distribution of the $^{211}$At tracer, although it can be imaged through the pinhole mode. There are several $\alpha$-emitting nuclides that can be used for RNT in the future^21,22^. The distribution of these nuclides should be comprehensible and controlled by monitoring characteristic X-rays and nuclear gamma rays from outside the body. Table 1 summarizes several properties of $\alpha$-emitting nuclides that are planned for clinical use. Moreover, X-ray or gamma-ray energy suitable for a specific imaging application may use different nuclides and consequently different energies as the 79 keV for $^{211}$At investigated here. Although some nuclides are easy to image with low-energy X-rays, others are suitable for gamma-ray imaging; for example, $^{225}$Ac and $^{212}$Bi. The wide scope of the hybrid cameras allows us to select the appropriate energy (from low-energy X-rays to high-energy gamma rays) and thus potentially cover a wide energy range including the conventional SPECT (140 keV), PET (511 keV) and Compton camera (sub-MeV to Multi-MeV) for various applications. This leads to a reduction in the measuring time, thus reducing the burden on the patient. In addition, wide-band imaging can image multiple tracers simultaneously, which significantly increases a patient’s complete medical information obtained from a single diagnosis.

To improve the performance for further applications, the hole size in the front detector should be adjusted as a trade-off between the efficiency and the resolution of pinhole imaging. Additionally, the application of the depth-of-interaction (DOI) technique^29–31^ to the backward detector may improve the resolution. Therefore, the energy range of the pinhole and Compton modes can be adjusted based on the density and/or thickness of the detectors. By revising the structure of the detector, imaging in a wider band can be realized. Particularly, Compton cameras developed for gamma-ray astronomy have realized imaging in high energy bands such as 1–10 MeV^32^. By applying the configuration of the hybrid camera as proposed in this paper, these Compton cameras can perform pinhole imaging without compromising on the performance of the original Compton camera. Currently, we are developing a new hybrid camera that covers 20 keV to 5 MeV by adopting the DOI configuration of the scintillator as described in Kishimoto et al. (2017)^14^ and Hosokoshi et al. (2019)^32^.

**Figure 7:**
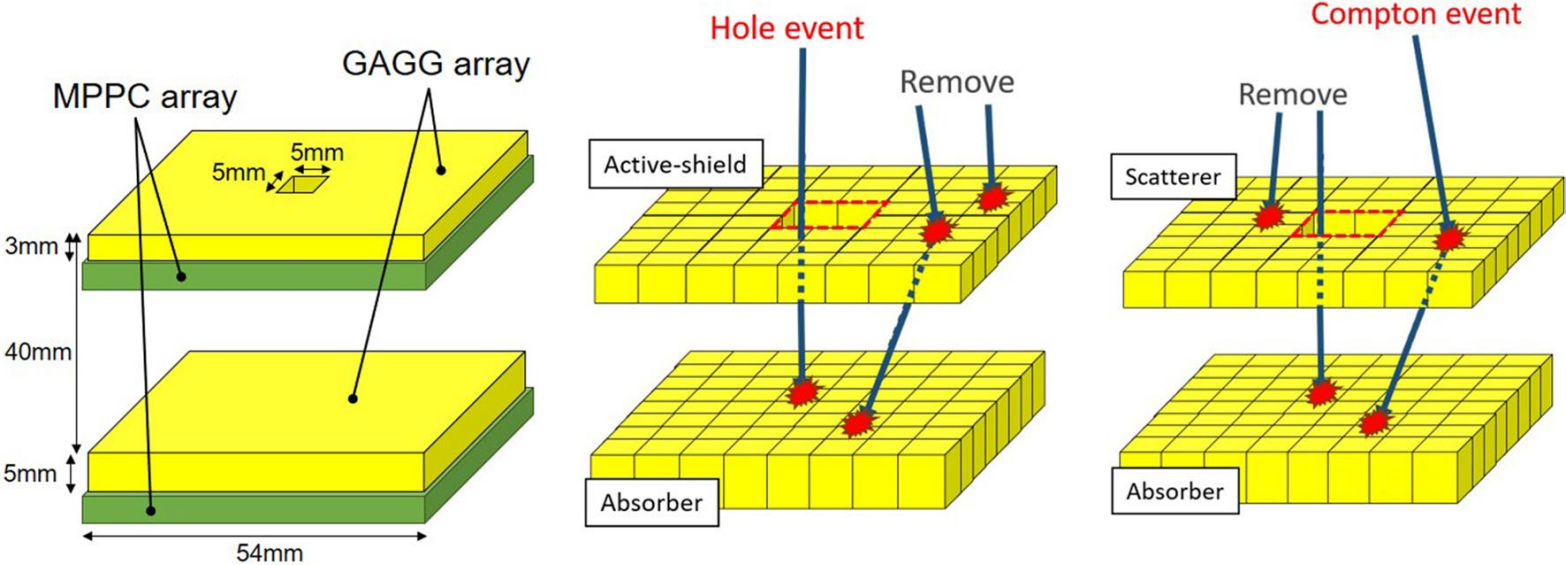
The configuration of the hybrid camera (left). Schematic view of the pinhole event (center) for the lower energy range and the Compton event (right) for the higher energy range that are used for the pinhole/Compton reconstruction in the hybrid camera.

## Methods

### Configuration of the hybrid camera

The configuration of the hybrid camera is detailed in Fig. 7 (left). The hybrid camera consists of a pair of position-sensitive detectors capable of receiving the reaction position and the energy deposit of each event. The detectors are composed of Ce-doped ${\mathrm{Gd}}_3{\mathrm{Al}}_2{\mathrm{Ga}}_3{\mathrm{O}}_{12}$ (GAGG) scintillator arrays^33,34^ coupled with multi-pixel photon counter (MPPC) arrays^29^. A $45\times 45$ array of $1 \times 1 \times 3$ mm$^3$ GAGG pixels is used as the front detector, and a $43 \times 43$ array of $1 \times 1 \times 5$ mm$^3$ GAGG pixels is used as the rear detector. The front detector has an active $5\times 5\, {\mathrm{mm}}^2$ pinhole in its center. The distance between the detectors is 4 cm. The energy resolutions of the front and rear detectors are 8.1% and 8.3% (FWHM) at 662 keV, respectively. The position resolution for gamma-ray interaction is equal to the pixel size, both for the pinhole and Compton mode. The time range of the coincidence is set to be ± 1 μs and the count rate capability is $\le$ 10 kHz. The camera is enclosed in a 3 mm-thick metal (mainly tungsten; density 18.0 ${\mathrm{g/cm}}^{3}$) case except the front surface.

The proposed hybrid camera enables imaging similar to that of a pinhole camera in addition to the conventional Compton camera. Commonly, the Compton camera, which consists of a scatterer and an absorber, uses events that undergo Compton scattering in the scatterer and photoabsorption in the absorber, as shown in Fig. 7 (right). For each event, the scattering angle of Compton scattering is calculated using Compton kinematics, restricting the arrival direction in the conical area called the Compton cone. The position of the radiation source is identified by superimposing the Compton cones. However, photons with energy lower than several hundreds of keV cannot be imaged by the Compton camera owing to the increased probability of photoabsorption in the front scatterer. The hybrid camera can also make use of such photoabsorption events. We have devised a method to operate the camera as a Compton and a pinhole camera by utilizing the front scatterer with a hole in the center. As shown in Fig. 7 (center), the arrival directions of low-energy photons are limited by analyzing the scatterer as an active pinhole. Among low-energy events detected in the rear detector, the events that are not detected in the front detector can be considered to have passed through the hole in the front detector. Note that Compton and/or pinhole reconstruction can be selected analytically after the measurement.

**Figure 8:**
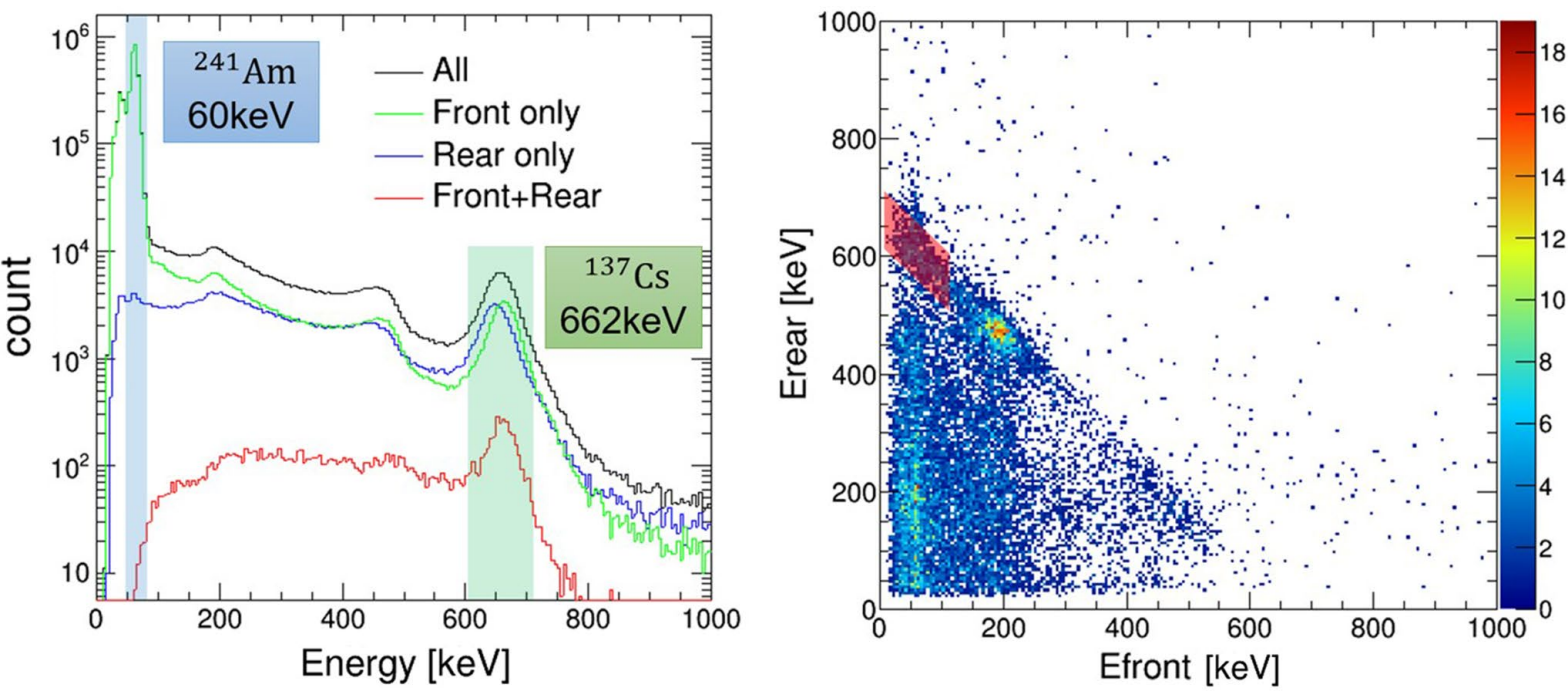
(Left) Energy spectrum of all the events detected in either detector (black), the events accumulated by with the front detector (green), the events obtained only with the rear detector (blue) and the events reacted with both detectors (red) accumulated from placing $^{241}$Am and $^{137}$Cs sources simultaneously in front of the detector. (Right) 2D energy spectrum of coincidence events from the front detector (scatterer) and the rear detector (absorber). The area painted red corresponds to the energy cut range for 662 keV Compton events. The brightest area ($E_{\mathrm{front}} \sim$ 200 keV) corresponds to back-scattering events.

**Table 2:** Factors of event selection for each reconstruction mode.

Mode	Coincidence selection	Energy cut
Pinhole	Anti-coincidence (rear)	Rear
Compton	Coincidence (front and rear)	Sum and front

**Table 3:** The numbers for applied energy cuts.

Radionuclide	Target (keV)	Mode	Energy cut (keV)
$^{241}$Am	60	Pinhole	$48< E_{\mathrm{rear}} < 72$
$^{137}$Cs	662	Compton	$E_{\mathrm{front}} < 110$, $612 < E_{\mathrm{front}}+E_{\mathrm{rear}} 712$
$^{211}$At	79	Pinhole	$69< E_{\mathrm{rear}} < 89$
$^{211}$At	570	Compton	$E_{\mathrm{front}} < 110$, $530< E_{\mathrm{front}}+E_{\mathrm{rear}} < 590$

### Reconstruction flow for each mode

The selection of valid events for Compton and pinhole modes are parameterized with two factors, coincidence selection and energy cut. Coincidence is an event pattern hitting one or both of the detectors. Specifically, only anti-coincidence events wherein only the rear detector is triggered are used for pinhole mode imaging, whereas coincidences between the front and rear detectors are used for Compton mode reconstruction. An energy cut is used to restrict the energy range of photons deposited in each detector. The event selection criteria for each mode are summarized in Table 2. From Fig. 8 (left, blue), the spectrum from the rear detector in the anti-coincidence mode simultaneously irradiated with $^{241}$Am and $^{137}$Cs sources shows that 60 keV X-ray is accurately masked in the front detector. Therefore, the pinhole events are selected by the energy cuts with the corresponding energy range. On the other hand, Compton events are chosen from candidates of coincident events. Figure 8 (left, red) shows the spectrum from coincidence events obtained with mixed $^{241}$Am and $^{137}$Cs measurement. Therefore, Compton events are chosen from the region of interest (red-diamond) restricted from the total energy deposit (612 keV $< E_{\mathrm{front}}+E_{\mathrm{rear}} < 712$ keV) and energy deposit of front detector to reject the back-scattering events ($E_{\mathrm{front}} < 110$ keV). Furthermore, the events whose scattering angles cannot be determined geometrically are deleted. A red diamond in the 2D spectrum of the coincidence events shown in Fig. 8 (right) corresponds to valid events for Compton image reconstruction. The quantitative numbers applied for energy cut are summarized in Table 3. In image reconstruction, maximum likelihood-expectation maximization (MLEM, a statistical approximation method)^15,35,36^ is used to improve the image quality. This method uses statistical iterations to locate sources with greater accuracy and better signal-to-noise ratio alternate to the simple back projection method. The number of iterations is 5 for each mode, except for the “L”-shaped Compton image, which is 15. In the pinhole mode, the signal-to-noise ratio is also improved by subtracting the background image that was reconstructed from the events next to the source energy range.
